# Treating Rett syndrome: from mouse models to human therapies

**DOI:** 10.1007/s00335-019-09793-5

**Published:** 2019-02-28

**Authors:** Neeti Vashi, Monica J. Justice

**Affiliations:** 1 Genetics and Genome Biology Program, The Hospital for Sick Children, The Peter Gilgan Centre for Research and Learning, Toronto, ON M5G 0A4 Canada; 2 Department of Molecular Genetics, University of Toronto, Toronto, ON M5S 1A1 Canada

## Abstract

Rare diseases are very difficult to study mechanistically and to develop therapies for because of the scarcity of patients. Here, the rare neuro-metabolic disorder Rett syndrome (RTT) is discussed as a prototype for precision medicine, demonstrating how mouse models have led to an understanding of the development of symptoms. RTT is caused by mutations in the X-linked gene methyl-CpG-binding protein 2 (*MECP2*). *Mecp2-*mutant mice are being used in preclinical studies that target the *MECP2* gene directly, or its downstream pathways. Importantly, this work may improve the health of RTT patients. Clinical presentation may vary widely among individuals based on their mutation, but also because of the degree of X chromosome inactivation and the presence of modifier genes. Because it is a complex disorder involving many organ systems, it is likely that recovery of RTT patients will involve a combination of treatments. Precision medicine is warranted to provide the best efficacy to individually treat RTT patients.

## Understanding and developing therapies for rare disorders

Rett syndrome (RTT) is a rare disorder that occurs in 1 in 10,000 females (Rett 1966; Haas 1988). It is characterized by seemingly normal neurological and physical development during the early postnatal period, followed by symptom manifestation between 6 and 18 months of age (Hagberg 2002). Symptoms progress over several stages (Table 1): stagnation, rapid regression, plateau, and late motor deterioration. The stagnation stage is characterized by subtle developmental delays in motor and language skills, and possible decreased alertness. This stage is often overlooked and leads to a delayed diagnosis, as parents and doctors may not notice these subtle changes. During the rapid regression stage, the child loses purposeful hand skills and spoken language, experiences motor impairments, and develops breathing abnormalities. Children may develop autistic-like features such as loss of interest in social interaction, and seizures may occur. This is followed by the plateau stage, during which motor problems and seizures become more common, but communication skills may improve. Lastly, children enter the late motor deterioration stage during which severe physical disability is common, and many patients become wheelchair dependent.

**Table 1:** Rett syndrome progresses over several stages

Stage	Age	Symptoms
1. Stagnation	6–18 months	• Developmental delays (postural control, motor, language) • Reduced eye contact • Hand-wringing may occur • Microcephaly may occur
2. Rapid regression	1–4 years	• Loss of purposeful hand skills • Stereotypical hand movements (wringing, washing, tapping) • Loss of spoken language • Walking may be unsteady • Breathing irregularities may occur • Autistic-like features • Microcephaly progression • Seizures may occur
3. Plateau/pseudo-stationary	2–potentially life	• Hand apraxia/dyspraxia • Motor coordination difficulties and/or loss of motor skills • Improvement of communication skills may occur • Seizures are common
4. Late motor deterioration	10–life	• Severe physical disability • Muscle weakness, rigidity, or spasticity • Wheelchair dependency may occur

Independent of disease stage, subsets of patients also experience gastrointestinal problems (Motil et al. 2012), abnormal cardiorespiratory coupling (Kumar et al. 2017), decreased bone density (Shapiro et al. 2010), early-onset osteoporosis (Haas et al. 1997), bruxism (Alpoz et al. 1999), dyslipidaemia (Justice et al. 2013; Segatto et al. 2014), inflammation of the gallbladder (Anderson et al. 2014), scoliosis (Anderson et al. 2014), urological dysfunction (Ward et al. 2016), and sleep disturbances (Young et al. 2007). Additionally, RTT patients have an increased incidence of unexpected death, and often die due to respiratory infection, cardiac instability, and respiratory failure (Laurvick et al. 2006; Anderson et al. 2014). Current treatment options are limited to symptom control.

## Rett syndrome is caused by mutations in methyl-CpG-binding protein 2 (*MECP2*)

Although a genetic basis for RTT was hypothesized as early as 1983 based on the preferential involvement of females (Hagberg et al. 1983), it was not until 1999 that a causative gene for the disorder was identified. Using a systematic gene screening approach, Amir et al. identified mutations in the gene methyl-CpG-binding protein 2 (*MECP2*) as the cause of some cases of RTT (Amir et al. 1999). It is now known that *de novo* mutations in *MECP2* account for 95% of typical RTT cases (Bienvenu et al. 2000). Although nearly 600 RTT-causing mutations have been identified in *MECP2*, only eight missense and nonsense mutations account for approximately 70% of mutations in RTT (R106W, R133C, T158M, R168X, R255X, R270X, R294X, and R306C) (Neul et al. 2008). Large deletions in *MECP2* account for another 15% of RTT-causing mutations. Interestingly, additional mutations in *MECP2* have been associated with autism (Xi et al. 2011), intellectual disability (Bianciardi et al. 2016), and lupus erythematosus (Liu et al. 2013).

*MECP2* encodes a nuclear protein (*MECP2*), which is especially abundant within neurons but is expressed at varying levels in every human tissue (Lewis et al. 1992; Tate et al. 1996; Shahbazian et al. 2002b). It is comprised of four domains and is host to several posttranslational modifications (Reviewed in Kyle et al. 2018). *MECP2* functions as a global transcriptional regulator by binding specifically to methylated DNA, recruiting protein partners and regulatory complexes to modify transcriptional activity (Nan et al. 1997; Chandler et al. 1999). Although *MECP2* is thought to primarily repress gene transcription, its role in transcriptional activation (Chahrour et al. 2008), chromatin remodeling, and mRNA splicing has also been described (reviewed in Lyst and Bird 2015). *MECP2* expression correlates with the postnatal maturation of the central nervous system (CNS) and neuronal differentiation, suggesting a role in CNS function and maintenance (Kishi and Macklis 2004). Within the brain, *MECP2* is seven times higher in neurons than in glia; however, *MECP2* has important roles in glia as well (Ballas et al. 2009; Skene et al. 2010; Lioy et al. 2011).

**Fig. 1:**
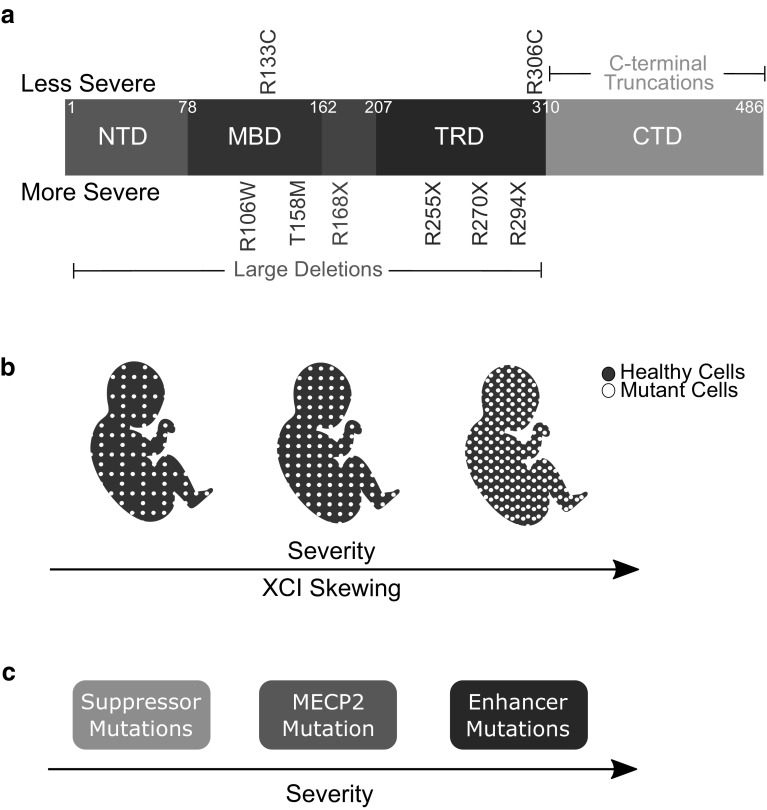
Symptom severity in RTT is influenced by mutation status, XCI pattern, and modifier genes. **a** Of the 8 most common RTT-causing *MECP2* mutations, R133C and R306C cause the least severe clinical presentation, whereas the missense mutations R106W and T158M, and nonsense mutations R168X, R255X, R270X, and R294X cause the most severe phenotype. Large deletions in the *MECP2* gene also cause a severe phenotype, whereas smaller C-terminal truncations are less severe. **b** Differences in XCI skewing patterns can influence clinical presentation, where patients with fewer cells expressing the mutant *MECP2* gene will have less severe symptoms. **c** Individuals who have modifier mutations in genes that suppress the RTT phenotype have a more favorable clinical presentation than individuals with mutations in genes that enhance detrimental symptoms

Clinical studies have highlighted the degree of phenotypic variation in Rett syndrome patients (Fig. 1a). Genotype–phenotype correlation studies demonstrate that early truncating mutations in *MECP2* (R168X, R255X, and R270X) and large INDELs cause the most severe phenotype, whereas most missense mutations (R133C and R306C) and late truncating mutations (R294X) are the mildest (Neul et al. 2008; Cuddapah et al. 2014). Thus, *MECP2* mutation status is a predictor of disease severity. Despite this, phenotypic variation is also reported in familial cases of RTT where affected sisters present with the same mutation (Zhang et al. 2017); these differences may be due to differences in X chromosome inactivation (XCI). Because *MECP2* is inherited on the X chromosome, female heterozygous RTT patients are mosaic carriers of normal and mutated *MECP2*. XCI is a random process by which one X chromosome is silenced in each cell. XCI can be skewed where the X chromosome carrying the mutated *MECP2* is more or less expressed throughout the brain and body, influencing the clinical presentation of RTT (Fig. 1b) (Ishii et al. 2001; Knudsen et al. 2006). Tests for skewed XCI are possible in the clinic. In cases where XCI is not skewed toward one allele, phenotypic variation may be due to the presence of modifier mutations. Modifiers are genes whose function has phenotypic outcomes on the effect of another gene. Mutations in modifier genes may alleviate or enhance clinical symptoms in patients as well (Fig. 1c).

## Mouse models recapitulate key symptoms of RTT

*Mecp2* is found in all vertebrates, but not in non-vertebrate genetic model organisms, including the fruit fly or the worm (Hendrich and Tweedie 2003). Therefore, developing mouse models of the disorder was needed for a mechanistic understanding of the onset and severity of clinical signs. Shortly after the identification of *MECP2* as the causative gene in RTT, two *Mecp2-*null mouse models were generated, which are now the primary models used to study the disease (Table 2). The *Mecp2*^*tm1.1Bird*^ mouse line completely lacks *MECP2* protein product (Guy et al. 2001), whereas the *Mecp2*^*tm1.1Jae*^ line expresses small *MECP2* protein fragments (Chen et al. 2001). However, both null models display a similar phenotype that recapitulates symptoms of RTT and both have been used extensively to study the mechanistic basis for disease.

**Table 2:** Many mouse models have been created to study Rett syndrome

Allele type	Allele	Description	Male phenotype							References
			RB	BW	BR	AX	M	PD	Age of death (weeks)	
**Null**										
*Mecp2* ^*tm1.1Bird*^	Null	Exon 3–4 deletion	X	X^	X	X	X	X	6–12	Guy et al. (2001)
*Mecp2* ^*tm1.1Jae*^	Null; some protein product retained	Exon 3 deletion	X	X*	X	X	X	X	10	Chen et al. (2001)
*Mecp2* ^*tm1Pplt*^	Null	MBD deletion	X	X	NT	X	X	X	8	Pelka et al. (2006)
**Human point mutations**										
*Mecp2* ^*tm4.1Joez*^	R106W	Missense mutation	X	NT	NT	NT	NT	X	10	Unpublished; MGI submission
*Mecp2* ^*tm1Nlnd*^	Y120D	Missense mutation	X	X	NT	–	X	X	14–17	Gandaglia et al. (2018)
*Mecp2* ^*tm6.1Bird*^	R133C	Missense mutation	X	X	NT	X	X	X	42	Brown et al. (2016)
*Mecp2* ^*tm1.1Joez*^	T158A	Missense mutation	X	–	NT	X	X	X	16	Goffin et al. (2011)
*Mecp2* ^*tm4.1Bird*^	T158M	Missense mutation	X	X	NT	X	X	X	13	Lyst et al. (2013), Brown et al. (2016)
*Mecp2* ^*tm3.1Joez*^	T158M	Missense mutation	X	NT	NT	NT	NT	X	14	Unpublished; MGI submission
*Mecp2* ^*tm1.1Jtc*^	R168X	Stop mutation; truncation	X	X	X	X	X	X	12–14	Lawson-Yuen et al. (2007), Schaevitz et al. (2013), Wegener et al. (2014)
*Mecp2* ^*tm1.1Irsf*^	R255X	Stop mutation; truncation	X	X	X	X	X	X	8–10	Pitcher et al. (2015)
*Mecp2* ^*tm5.1Bird*^	R306C	Missense mutation	X	X	NT	X	X	X	30	Lyst et al. (2013), Brown et al. (2016)
**Other mutations**										
*Mecp2* ^*tm2.1Jae*^	S80A	Missense mutation	NT	X*	NT	NT	X	NT	NT	Tao et al. (2009)
*Mecp2* ^*tm1Vnar*^	A140V	Missense mutation	–	–	–	–	–	–	Normal lifespan	Jentarra et al. (2010)
*Mecp2* ^*tm3Meg*^	T308A	Missense mutation	X	NT	NT	NT	X	NT	> 16	Ebert et al. (2013)
*Mecp2* ^*tm1Hzo*^	R308X	Stop mutation; truncation	X	–	NT	X*	X	X	6–12	Shahbazian et al. (2002a)
*Mecp2* ^*tm1.1Meg*^	S421A	Missense mutation	–	–	–	–	–	–	Normal lifespan	Cohen et al. (2011)
*Mecp2* ^*tm1.1Mitoh*^	Deletion	Isoform 2 deletion	–	–	–	–	–	–	Normal lifespan	Itoh et al. (2012)
*Mecp2* ^*tm1.1Dhy*^	Deletion	Isoform 1 deletion	X	–	NT	X	X	X	7–31	Yasui et al. (2014)
**Conditional alleles**										
*Mecp2* ^*tm1Bird*^	–	Exons 3–4 floxed	X*	–	X	X	X	–	Normal lifespan	Guy et al. (2001), Samaco et al. (2008)
*Mecp2* ^*tm1Jae*^	–	Exon 3 floxed	–	–	–	–	–	–	Normal lifespan	Chen et al. (2001)
*Mecp2* ^*tm2Bird*^	–	Floxed-stop upstream of exon 3	X	X	X	X	X	X	8–10 weeks	Guy et al. (2007)

These mouse models include null alleles, point mutations designed to recapitulate mutations observed in human RTT patients, whole exon deletions, and conditional alleles used in combination with targeted Cre mice to achieve temporal deletion of *Mecp2*. In BW category, alleles marked with *which have an increased body weight, and alleles marked with ^show either increase or decrease depending on mouse background. In the AX category, alleles marked with *show increased anxiety

*RB* reduced brain size, *BW* body weight reduction, *BR* breathing abnormalities, *AX* reduced anxiety, *MA* motor abnormalities, *PD* premature death In categories, *X* present, – not present, *NT* not tested

RTT predominantly affects females, so female *Mecp2-*mutant mice are the most clinically relevant model. However, female *Mecp2-*mutant mice display deviations in phenotype presentation due to XCI skewing making it difficult to discern which phenotypes arise through cell autonomous versus non-autonomous pathways. Therefore, the majority of RTT mouse studies take place in hemizygous *Mecp2-*mutant male mice due to their consistent phenotype and their complete lack of *MECP2* protein, which is advantageous for mechanistic research.

Hemizygous male *Mecp2*-null mice are phenotypically normal until 4 weeks of age when they develop a RTT-like phenotype consisting of hind limb clasping, tremors, breathing irregularities, loss of muscle tone, and hypoactivity (Chen et al. 2001; Guy et al. 2001). These mice also display a reduced brain weight and body weight, experience a rapid phenotypic regression, and die between 6 and 12 weeks of age. Female mice heterogeneous for *Mecp2* deletions develop the same features at 4–6 months of age and typically live a normal lifespan.

**Fig. 2:**
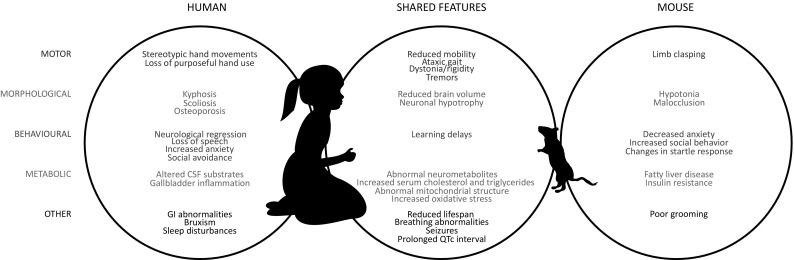
RTT patients and *Mecp2*-mutant mouse models share many features

*Mecp2-*mutant mouse models recapitulate a broad spectrum of phenotypes seen in human RTT patients (Fig. 2). The most profound symptoms in human patients and most *Mecp2-*mutant mice are motor abnormalities, including reduced mobility, impaired motor coordination, ataxic gait, and tremors (Hagberg and Witt-Engerström 1986). While human patients replace purposeful hand use with stereotypic movements, mouse models clasp their hindlimbs (Chen et al. 2001; Guy et al. 2001); this may resemble the hallmark human symptom, but it is not a phenotype specific to RTT mouse models (Lalonde and Strazielle 2011). Morphologically, both human patients and *Mecp2* mouse models exhibit a reduced brain volume and neuronal hypotrophy (Chahrour and Zoghbi 2007). Behaviorally, RTT patients experience a neurological regression and loss of speech, two traits which cannot be measured in mouse models. However, both may experience some learning deficits (Elefant and Wigram 2005; Moretti 2006). Interestingly, while human RTT patients typically exhibit increased anxiety and social avoidance (Mount et al. 2002), *Mecp2*-mutant mice are less anxious and more social, as assessed by phenotypic tests available for rodents (Samaco et al. 2012; Orefice et al. 2016; Wu et al. 2016). Metabolic disturbances in both human patients and mice include abnormalities in neurometabolites (Goffin and Zhou 2012), increased serum cholesterol and triglycerides (Buchovecky et al. 2013; Justice et al. 2013; Segatto et al. 2014; Kyle et al. 2018), abnormal mitochondrial structure (Shulyakova et al. 2017), and increased oxidative stress (De Felice et al. 2009; Janc et al. 2016). Finally, both experience breathing irregularities (Ramirez et al. 2013), cardiac abnormalities (prolonged QTc intervals) (McCauley et al. 2011; Hara et al. 2015), seizures, and a shortened lifespan (Guy et al. 2001; Anderson et al. 2014). Overall, *Mecp2-*mutant mice exhibit a broad range of phenotypes that recapitulate symptoms of human RTT patients, making them excellent models to study the disorder.

Despite their face validity, null mouse models do not always represent human cases molecularly since many RTT patients carry missense mutations that result in a less-efficient or unstable *MECP2* protein rather than a complete loss of it. To circumvent this caveat, several mouse lines with point mutations and deletions in *MECP2* have been engineered to recapitulate clinically relevant mutations observed in human patients (Table 2). Of the eight most common missense mutations in RTT patients, six have an established mouse model which recapitulates symptoms of the disease. These mice have been instrumental in understanding the functional consequences of RTT-causing mutations and their correlation to symptom severity. For example, RTT patients carrying *MECP2* T158M mutations display severe RTT symptoms, while motor and speech preservation is observed in patients with a R133C mutation (Cuddapah et al. 2014). Consistently, male mice carrying these missense mutations have a lifespan of 13 weeks and 42 weeks, respectively. Functional studies in mice determined that while both the T158M and R133C mutations create *MECP2* proteins with reduced DNA-binding ability, the T158M mutation also leads to protein instability, likely resulting in the unique and more severe phenotype presentation (Brown et al. 2016).

Further, mice with *Mecp2* mutations have been designed to study specific biological questions of functional relevance. For example, while *MECP2* is expressed as two isoforms, their expression levels vary among tissues, with *MECP2*_e1 being more abundant than e2 in the brain. Studies that deleted either *MECP2*_e1 or e2 throughout the body found that only deletion of *MECP2*_e1 led to RTT-like phenotypes and a shortened lifespan, while deletion of e2 was required for placental development (Itoh et al. 2012; Yasui et al. 2014). Additionally, *MECP2* protein is a host to numerous posttranslational modifications (PTMs), including phosphorylation at serine 80 (S80) and 421 (S421). Mutating either of these serine residues in mice prevented this site-dependent phosphorylation and determined that these PTMs are important for the association of *MECP2* to chromatin and its role in regulating neuronal gene expression (Tao et al. 2009; Cohen et al. 2011).

Finally, conditional-ready alleles have been constructed by engineering *lox*P sites flanking a portion of the *Mecp2* gene (Floxed) (Chen et al. 2001; Guy et al. 2001). These mice can be crossed with transgenic mice carrying a tissue- or cell type-specific Cre to achieve spatial or temporal deletion of *Mecp2*. Studies using these mice have greatly informed *Mecp2* function both within the CNS and in peripheral tissues. Neuronal dysfunction was once considered the only cause of RTT, despite *MECP2*’s expression in glial cells. Crossing *Mecp2-*floxed mice to mice with a glial-specific *GFAP*-Cre transgene achieved targeted deletion of *Mecp2* in astrocytes. These mice developed RTT-like symptoms including decreased body weight, hindlimb clasping, and irregular breathing, demonstrating that glial *Mecp2* deficiency could produce some RTT symptoms (Lioy et al. 2011). Additionally, liver-targeted deletion of *Mecp2* was achieved by crossing *Mecp2-*floxed mice to mice expressing Cre under the control of an *Albumin* promoter (*Alb*-Cre). The resulting mice develop fatty liver disease and dyslipidemia, implicating *MECP2* in the regulation of lipid metabolism in non-CNS tissues (Kyle et al. 2016). Since transgenic mice have been engineered to express Cre from many diverse cell types, continued studies are likely to achieve a thorough understanding of *Mecp2’s* role in a variety of tissues and cell types.

A significant question in RTT research concerns phenotypic reversibility: Since RTT patients do not display neuronal death, it is possible that *Mecp2-*deficient cells can be repaired. In a milestone study, a mouse model was engineered to contain a transcriptional STOP cassette flanked by *lox*P, which was inserted into the endogenous *Mecp2* gene (Guy et al. 2007). These ‘*FloxedStop’* mice were crossed with mice expressing a ubiquitous Cre-ER transgene. This strategy allowed for *Mecp2* to remain silenced in mice until Cre could be activated with injection of tamoxifen. ‘*FloxedStop’* male mice were identical to *Mecp2-*null mice, developed RTT-like symptoms and had a shortened lifespan. Following phenotype onset, tamoxifen was administered systemically, removing the *loxP-*STOP-*loxP* cassette and restoring *Mecp2* expression to 80% of normal levels. Remarkably, restoring *MECP2* expression reversed neurological symptoms and normalized lifespan (Guy et al. 2007). This key study demonstrated that *Mecp2-*induced pathology is not permanent and symptom reversal may be possible in human patients.

**Fig. 3:**
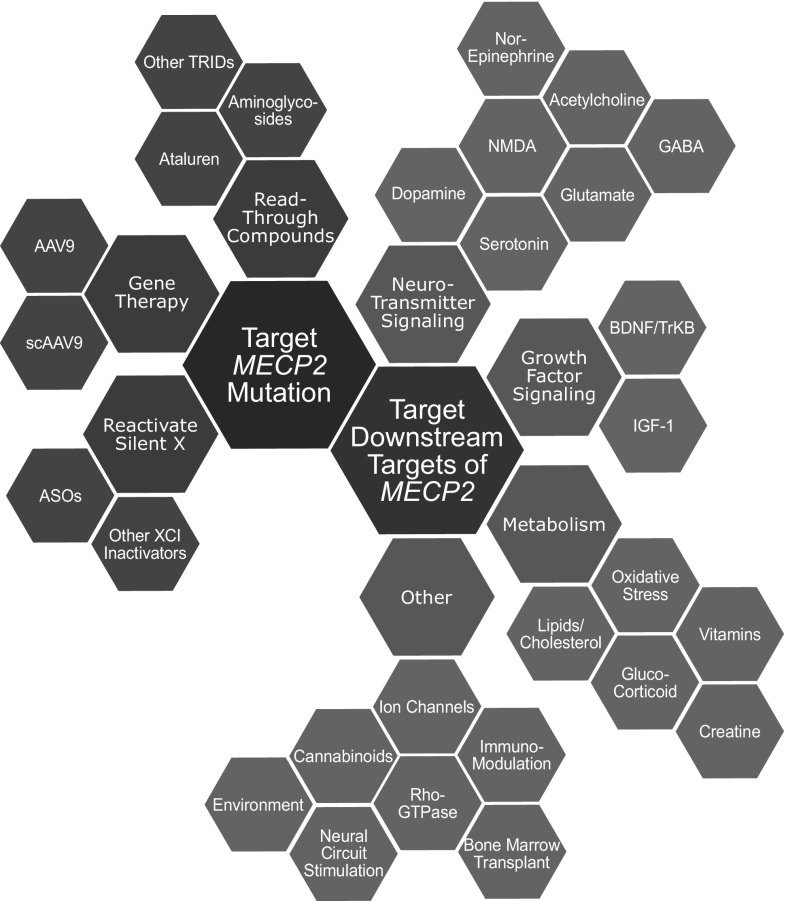
Treatment options in RTT either directly target *MECP2* mutation or target pathways downstream of *MECP2*. Treatment options are further divided within these two groups

Given *MECP2*’s high expression levels, its global DNA-binding affinity, and its multitude of proposed binding partners and functions, it is unsurprising that *Mecp2-*deficiency results in a myriad of dysregulated pathways. *MECP2* has been implicated in neuronal maintenance (Kishi and Macklis 2004; Guy et al. 2011), glial cell function (Ballas et al. 2009), neurotransmitter signaling (Goffin and Zhou 2012), growth factor signaling (Itoh et al. 2007), lipid metabolism (Buchovecky et al. 2013), oxidative stress (Großer et al. 2012), and more. Current preclinical research follows two approaches: targeting pathways downstream of *MECP2* or directly targeting *MECP2* and its protein product (Fig. 3).

## Using mouse models to develop therapeutics for RTT targeting pathways downstream of *Mecp2*

The first approach of recognizing components that lie in pathways downstream of *MECP2* has identified many targets with the potential for treatment. Pharmacological therapeutics for targets downstream of *MECP2* can be repurposed to improve neurotransmitter signaling, growth factor signaling, and metabolism (Table 3). Importantly, several of these treatments ameliorated symptoms in *Mecp2-*mutant mice and have spawned clinical trials in patients. However, these strategies revealed several limitations. First, because the precise functions of *MECP2* are still unknown, identifying the pathways it regulates, especially those which may be subtly changed in the absence of *MECP2*, is difficult. Another challenge is specificity: *MECP2* is recruited to DNA methylation signals, which are highly cell-specific (Cedar 1988; Deaton et al. 2011). Therefore, the genes *MECP2* regulates will likely vary based on methylation status in different cell types. Consistently, a recent study found that gene misregulation within subtypes of neurons in *Mecp2* mutant mice is highly dependent on cell type-specific epigenetic marks (Renthal et al. 2018). Finally, it is difficult to discern which pathways are affected as the primary result of *MECP2* loss and which are secondary effects farther downstream that will likely have less value as therapeutic targets. Nevertheless, advances in molecular biology techniques are making it easier to circumvent these issues. For example, single-cell RNA sequencing can identify genes misregulated in individual *Mecp2*-deficient cells and chromatin immunoprecipitation of *MECP2*’s known binding partners could identify its direct transcriptional targets in individual cells. Here, we highlight preclinical treatments targeting pathways downstream of *Mecp2* which have led to clinical trials in RTT patients.

**Table 3:** Preclinical treatments targeting pathways downstream of *Mecp2*

Treatment	Mechanism	Mouse model	Prolong lifespan	Improved phenotype	References	Clinical trial
**Neurotransmitter signaling**						
Citalopram	Serotonin reuptake blocker	*Mecp2* ^*tm1.1Bird*^	NT	Improved sensitivity to carbon dioxide exposure	Toward et al. (2013)	–
8-OH-DPAT	Serotonin 1a agonist	*Mecp2* ^*tm1.1Bird*^	NT	Reduced apneas	Abdala et al. (2010)	–
F15599	Serotonin 1a agonist	*Mecp2* ^*tm1.1Bird*^	NT	Reduced apneas and improves breathing irregularity	Levitt et al. (2013)	–
Sarizotan	Serotonin 1a agonist & dopamine D2-like receptor	*Mecp2* ^*tm1.1Bird*^ *Mecp2* ^*tm1.1Jae*^ *Mecp2* ^*tm1.1Jtc*^	NT	Reduced apneas and improved breathing irregularity	Abdala et al. (2014)	Y
LP-211	Serotonin 7 receptor agonist	*Mecp2* ^*tm1Hzo*^	NT	Improved overall health, memory and anxiety	De Filippis et al. (2014)	–
Levodopa	Dopaminergic stimulation	*Mecp2* ^*tm1.1Bird*^	Y	Improved motor activity	Szczesna et al. (2014)	–
Ketamine	NMDA receptor antagonist	*Mecp2* ^*tm1.1Jae*^	NT	Improved startle response	Kron et al. (2012)	Y
Ketamine	NMDA receptor antagonist	*Mecp2* ^*tm1.1Bird*^	Y	Improved limb clasping, motor coordination and reduced apneas	Patrizi et al. (2016)	Y
NO-711	GABA reuptake blocker	*Mecp2* ^*tm1.1Bird*^	NT	Reduced apneas	Abdala et al. (2010)	–
Benzodiazepine diazepam	GABA reuptake blocker	*Mecp2* ^*tm1.1Bird*^	NT	Reduced apneas	Abdala et al. (2010)	–
L-838,417	GABA reuptake blocker	*Mecp2* ^*tm1.1Bird*^	NT	Reduced apneas	(Abdala et al. 2010)	–
Tiagabine	GABA reuptake blocker	*Mecp2* ^*tm1.1Bird*^	Y	No improvement	El-Khoury et al. (2014)	–
THIP	GABA receptor agonist	*Mecp2* ^*tm1.1Bird*^	Y	Improved motor function, social behavior, and reduced apneas	Zhong et al. (2016)	–
Mirtazapine	GABA release promoter	*Mecp2* ^*tm1.1Bird*^	NT	Improved overall healthy, neuronal morphology, dendritic spine number, anxiety	Bittolo et al. (2016)	–
VUO462807	mGlu_5_ positive allosteric modulator	*Mecp2* ^*tm1.1Bird*^	N	Improved motor function and limb clasping	Gogliotti et al. (2016)	–
VU0422288	mGlu_7_ positive allosteric modulator	*Mecp2* ^*tm1.1Bird*^	NT	Reduced apneas, improves learning and memory	Gogliotti et al. (2017)	–
CTEP	mGluR_5_ negative allosteric modulator	*Mecp2* ^*tm1.1Bird*^	Y	Reduced apneas, improved memory	Tao et al. (2016)	–
Acetyl-L-carnitine	Acetyl group donor	*Mecp2* ^*tm1.1Jae*^	NT	Improved weight gain, motor activity and memory	Schaevitz et al. (2012)	–
Acetyl-L-carnitine	Acetyl group donor	*Mecp2* ^*tm1.1Jae*^	NT	Improved weight gain, motor activity and memory	Schaevitz et al. (2012)	–
Choline	ACh	*Mecp2* ^*tm1.1Jae*^	NT	Improved motor coordination and activity	Nag and Berger-Sweeney (2007)	–
Choline	ACh	*Mecp2* ^*tm1Hzo*^	NT	Improved motor activity	Ricceri et al. (2011)	–
Choline	ACh	*Mecp2* ^*tm1.1Bird*^	NT	Improved motor coordination, anxiety and social behavior	Chin et al. (2018)	–
D-NAC	Dendrimer-conjugated N-acetyl cysteine	*Mecp2* ^*tm1.1Bird*^	NT	Improved overall health, limb clasping	Nance et al. (2017)	–
Desipramine	Norepinephrine reuptake inhibitor	*Mecp2* ^*tm1.1Bird*^	Y	Improved breathing irregularities	Roux et al. (2007)	Y
Desipramine	Norepinephrine reuptake inhibitor	*Mecp2* ^*tm1.1Bird*^	Y	Reduced apneas	Zanella et al. (2008)	Y
Clenbuterol	B2-adrenergic receptor agonist	*Mecp2* ^*tm1.1Bird*^	Y	Improved motor coordination and breathing irregularities	Mellios et al. (2014)	–
D-cycloserine	D-alanine analog	*Mecp2* ^*tm1.1Jae*^	NT	No improvement	Na et al. (2017)	–
**Growth factor signaling**						
CX546	Ampakine (BDNF)	*Mecp2* ^*tm1.1Jae*^	NT	Improved breathing irregularity	Ogier et al. (2007)	–
Fingolimod	Sphingosine-1 phosphate receptor (BDNF)	*Mecp2* ^*tm1.1Bird*^	NT	Improved motor activity	Deogracias et al. (2012)	Y
CPT157633	PTP1B inhibitor (BDNF)	*Mecp2* ^*tm1.1Bird*^	Y	Reduced limb clasping, partially improved motor activity	Krishnan et al. (2015)	–
LM22A-4	TrkB agonist (BDNF)	*Mecp2* ^*tm1.1Jae*^	NT	Improved breathing irregularity	Schmid et al. (2012)	–
7,8-DHF	TrKB agonist (BDNF)	*Mecp2* ^*tm1.1Jae*^	Y	Improved motor activity and breathing irregularities	Johnson et al. (2012)	–
LM22A-4	TrkB agonist (BDNF)	*Mecp2* ^*tm1.1Jae*^	NT	Reduced apneas	Kron et al. (2014)	–
LM22A-4	TrkB agonist (BDNF)	*Mecp2* ^*tm1.1Jae*^	NT	Improved memory	Li et al. (2017)	–
IGF-1	IGF-1	*Mecp2* ^*tm1.1Jae*^	Y	Improves motor activity, breathing irregularities, increased brain size	Tropea et al. (2009)	Y
PEG-IGF-1	Slow release IGF-1	*Mecp2* ^*tm1.1Bird*^	Y	No improvement	Pitcher et al. (2013)	–
RhIGF01	Recombinant human IGF1-1	*Mecp2* ^*tm1.1Bird*^	Y	Improves motor activity, breathing irregularities, social behavior and anxiety	Castro et al. (2014)	Y
**Metabolism**						
Diet restriction	Caloric deficit	*Mecp2* ^*tm1Hzo*^	NT	Improved motor activity and anxiety	Mantis et al. (2009)	–
Statins	Cholesterol-lowering medication	*Mecp2* ^*tm1.1Bird*^ *Mecp2* ^*tm1.1Jae*^	Y	Improved overall health, motor coordination, motor activity, serum lipids and liver lipids	Buchovecky et al. (2013)	Y
Dietary triheptanoin	Energy use (mitochondria)	*Mecp2* ^*tm1.1Jae*^	Y	Improved motor coordination, social behavior, insulin sensitivity, metabolic homeostasis	Park et al. (2014)	Y
Trolox	Vitamin E derivative	*Mecp2* ^*tm1.1Bird*^	NT	Blood glucose levels normalized, improved response to hypoxia	Janc et al. (2016)	–
Corticosterone	Glucocorticoid activation	*Mecp2* ^*tm1.1Bird*^	Decreased lifespan	Worsened motor activity	Braun et al. (2012)	–
Corticosterone	Glucocorticoid activation	*Mecp2* ^*tm1Hzo*^	NT	Improved motor activity	De Filippis et al. (2013)	–
RU486	Glucocorticoid repression	*Mecp2* ^*tm1.1Bird*^	No	Delayed progression of symptoms, improved motor activity	Braun et al. (2012)	–
Curcumin	Anti-oxidant, anti-inflammatory	*Mecp2* ^*tm1.1Jae*^	NT	NT	Panighini et al. (2013)	–
Insulin	Glucose signaling	*Mecp2* ^*tm1.1Bird*^	Decreased lifespan	No improvement	Pitcher et al. (2013)	–
**Other**						
Zoledronic acid	Anti-osteoclastic	*Mecp2* ^*tm1.1Bird*^	NT	Increased bone volume and connectivity	Shapiro et al. (2010)	–
Cannabidivarin	Phytocannabinoid	*Mecp2* ^*tm1Hzo*^	NT	Improved overall health, motor activity and social behavior	Vigli et al. (2018)	–
CNF1	RhoGTPase	*Mecp2* ^*tm1Hzo*^	NT	Improved motor activity	De Filippis et al. (2012)	–
CNF1	Rho GTPase	*Mecp2* ^*tm1Hzo*^	NT	Improved mitochondrial dysfunction and memory	De Filippis et al. (2015)	–
**Non-pharmacological**						
Enriched environment	Environmental modulation	*Mecp2* ^*tm1Pplt*^	NT	Improved motor coordination	Kondo et al. (2008)	–
Enriched environment	Environmental modulation	*Mecp2* ^*tm1.1Jae*^	NT	Improved motor activity	Nag et al. (2009)	–
Enriched environment	Environmental modulation	*Mecp2* ^*tm1.1Jae*^	N	Improved motor coordination and activity	Lonetti et al. (2010)	–
Enriched environment	Environmental modulation	*Mecp2* ^*tm1Pplt*^	NT	Reduced anxiety	Kondo et al. (2016)	–
Forniceal deep brain stimulation	Neural circuit stimulation	*Mecp2* ^*tm1.1Bird*^	NT	Improved memory	Hao et al. (2015)	–
Bone marrow transplantation	Brain microglia repopulation	*Mecp2* ^*tm1.1Bird*^	Y	Reduced apneas, improved breathing irregularities, improved locomotor activity	Derecki et al. (2012)	–

Treatment strategies are divided into those influencing neurotransmitter signaling, growth factor signaling, metabolism, other pharmacological treatments, and non-pharmacological treatments. In prolong lifespan column, *Y* yes, *N* no, *NT* not tested. In clinical trial column, *Y* yes, –: not yet tested

### Potential treatments targeting neurotransmitter signaling

A prominent feature of RTT and *Mecp2*-mutant mouse models is the reduction in the number and length of dendrites (Armstrong 1992). Since dendrites receive electrical signals and neurotransmitters from pre-synaptic neurons, a neurotransmitter signaling defect was proposed in RTT. In support of this idea, dysfunction in dopamine (Wenk 1995), serotonin (Paterson et al. 2005; Ohno et al. 2016), norepinephrine (Viemari 2005; Santos et al. 2010), glutamate (Chao et al. 2010; Abdala et al. 2016), and NMDA signaling (Katz et al. 2016; Patrizi et al. 2016) has been observed in *Mecp2*-mutant mice. Treatments that target these pathways have shown varied effects in mice, and a few have been tested in clinical trials. Desipramine, a norepinephrine reuptake inhibitor, improved breathing irregularities and apneas in *Mecp2-*mutant mice (Roux et al. 2007; Zanella et al. 2008). However, a clinical trial found no clinical improvements in RTT patients treated with this drug (Mancini et al. 2018). In contrast, sarizotan, a serotonin 1a agonist and dopamine D2-like receptor, reduced breathing apneas by 15–30% in *Mecp2-*mutant mice but had no effect on motor activity (Abdala et al. 2014). As a result, it is currently being tested for its efficacy in improving respiratory symptoms in RTT (NCT02790034).

Finally, ketamine, an NMDA receptor agonist, was tested in two different laboratories for its therapeutic potential in RTT (Kron et al. 2012; Patrizi et al. 2016). These preclinical studies showed that low-dose ketamine could increase activity in the cortical network while decreasing synaptic excitability in the brainstem network, targeting a possible imbalance in neuronal activity throughout the *Mecp2-*mutant brain. Treatment with ketamine improved limb clasping, motor coordination, and breathing apneas in *Mecp2-*mutant mice. The safety of ketamine is currently being assessed in RTT patients (NCT03633058).

### Growth factor signaling as a treatment for RTT

Early studies of *MECP2* identified BDNF as one of its transcriptional targets (Chen et al. 2003). BDNF is a member of the neurotrophin family of growth factors, which binds to tropomyosin-related kinase B (TrkB) to stimulate signaling cascades involved in neurite outgrowth, synaptic function, and neuronal differentiation (Amaral and Pozzo-Miller 2007). In RTT patients and *Mecp2-*mutant mice, brain *Bdnf* expression is drastically reduced (Chang et al. 2006; Deng et al. 2007). However, direct administration of BDNF is not a feasible treatment option because it is unable to cross the blood–brain barrier (BBB). However, fingolimod (FTY720), a sphingosine-1 phosphate analog, has been shown to partially increase BDNF levels and improve motor activity in *Mecp2-*mutant mice (Deogracias et al. 2012). Fingolimod is currently being assessed for its safety and efficacy in patients (NCT02061137).

Insulin-like growth factor-1 (IGF) binds to the IGF-1 receptor (IGF-1R), activating a signaling cascade similar to the BDNF receptor TrkB. IGF-1 is also transcriptionally regulated by *MECP2* (Itoh et al. 2007), but unlike BDNF, it can cross the BBB, increasing its therapeutic potential (Baker et al. 2005). IGF-1 signaling is involved in neuronal survival, neural outgrowth, and synapse formation (D’Ercole et al. 1996). It has been implicated as a promising therapeutic in cancer, diabetes, and ALS.

*Mecp2-*mutant mice treated with IGF-1 showed improved locomotor function, breathing irregularities, and extended lifespan. IGF-1 also increased the brain weight of mutant mice while restoring neural spine density in the motor cortex and improving excitatory synaptic transmission in sensorimotor cortex neurons (Tropea et al. 2009). Importantly, full-length IGF-1 is already an approved treatment in North America used to treat growth failure in children. A phase 1 clinical trial in RTT patients found no adverse effects of recombinant human IGF-1 (rhIGF-1) (Khwaja et al. 2014). However, subsequent clinical trials found it produced no significant improvement of RTT symptoms (O’Leary et al. 2018).

Recently, a synthetic analog derived from IGF-1, NNZ-2566 (Trofinetide) was developed which inhibits neuroinflammation, restores glial function, corrects synaptic deficits, and regulates oxidative stress response (Deacon et al. 2015). When administered to mice with Fragile X syndrome, NNZ-2566 drastically improved their aberrant symptoms (Ligsay and Hagerman 2016). A phase 2 clinical trial of NNZ-2566 in RTT patients showed that treatment improved overall health, neurobehavioral and motor symptoms. These promising results enabled a phase 3 clinical trial that is currently recruiting patients (NCT02715115).

### Metabolic defects in RTT can be therapeutically targeted

Modifiers of *MECP2* have implicated neurite outgrowth and energy homeostasis in altering phenotypic outcome in human patients (Artuso et al. 2011; Pizzo et al. 2018). Modifiers are also being identified and validated by carrying out modifier screens in a mouse model (Buchovecky et al. 2013). In a modifier screen, unbiased random mutation identifies key genes that can change a phenotype of interest, allowing the model to reveal important pathways of interest. Such an approach was applied to identify novel therapeutic targets in RTT; ethylnitrosourea (ENU), a mouse supermutagen, was used to induce mutations in male mice, which were then bred to females carrying a mutant *Mecp2*. Male mice inheriting mutant *Mecp2* would normally become very ill and die. Dominant second site mutations that could improve the health and neurological phenotype of the *Mecp2* mutant males were then identified molecularly (Buchovecky et al. 2013). One suppressor of disease was a nonsense mutation in a rate-limiting enzyme in cholesterol synthesis—a pathway that had not previously been associated with RTT.

The identification of this modifier locus led to additional studies that revealed lipid metabolism was severely perturbed in *Mecp2*-mutant mice. Prior to symptom onset, brain cholesterol was already markedly elevated in *Mecp2-*mutant mice, and cholesterol and triglycerides were elevated in the serum and liver. Remarkably, lipid-lowering statin drugs regulated lipid levels, ameliorated motor symptoms, and extended lifespan in mice (Buchovecky et al. 2013). Furthermore, elevated lipids have also been observed in a subset of RTT patients, indicating that repurposing of statin drugs may be a viable treatment option to benefit patients (Justice et al. 2013; Segatto et al. 2014). Abnormalities in cholesterol homeostasis are associated with many neurological diseases (Tint et al. 1994; Puglielli et al. 2003; Bi and Liao 2010; Berry-Kravis et al. 2015), and therefore RTT is no exception. Importantly, these findings led to a clinical trial testing the efficacy and safety of statins in RTT patients (NCT02563860).

Metabolic defects in RTT are not limited to cholesterol synthesis. Both RTT patients and *Mecp2-*mutant mouse models display abnormal mitochondrial structure and function (Eeg-Olofsson et al. 1988; Dotti et al. 1993; Kriaucionis et al. 2006; Gold et al. 2014). Mitochondria are organelles within cells that convert glucose into adenosine triphosphate (ATP), the energy currency of the cell. Importantly, compromised mitochondrial function can greatly affect cellular energy production. While mitochondria are necessary in all cells of the body, their role is especially important in tissues with high energy demands, such as nerves and muscle. RTT shares many features of mitochondrial diseases, including early symptomatic onset, developmental delay, neurological regression, poor muscle tone, seizures, and gastrointestinal issues (Schon and Manfredi 2003). Consistently, both RTT patients and *Mecp2-*mutant mice present with increased oxidative stress and decreased levels of mitochondrial enzymes (Haas et al. 1995; Kriaucionis et al. 2006; De Felice et al. 2009; Leoncini et al. 2011). In *Mecp2-*mutant mice, markers of oxidative stress increase with age, suggesting a progressive dysfunction in mitochondrial function (De Felice et al. 2014). Altogether, this suggests defects in mitochondrial energy production may be present in RTT. Importantly, mitochondrial energy production is tightly linked with cholesterol synthesis. Several steps in the biosynthesis of cholesterol require mitochondrial sources of ATP as an electron donor in oxygenation reactions. Thus, cholesterol perturbations may be linked with mitochondrial dysfunction in RTT.

Interestingly, anaplerotic substances can replenish intermediate compounds in the energy production pathway, enhancing mitochondrial energy production. A diet supplemented with the anaplerotic triheptanoin reversed symptoms of some metabolic disorders by correcting energy production (Roe et al. 2002; Mochel et al. 2005). This strategy was adapted for use in mouse models of RTT. Strikingly, *Mecp2*-mutant mice fed a diet with triheptanoin supplementation showed improved mitochondrial morphology and improved energy production. This translated to improved motor coordination and increased lifespan in these mice. Two clinical trials investigating the efficacy of triheptanoin in improving seizures, muscle tone, and symptom improvement in RTT patients are currently in their early stages (NCT02696044 and NCT03059160).

## Using mouse models to develop therapies for RTT that directly target *MECP2*

Directly restoring *MECP2* is an attractive therapeutic strategy (Fig. 3) since all pathways downstream of *MECP2* could subsequently recover as well. However, the largest concern of these approaches is dosage. While lack of *MECP2* causes RTT, an abundance of *MECP2* causes *MECP2* duplication syndrome, a disorder characterized by neurological dysfunction, intellectual disability, and some RTT-like features (Van Esch et al. 2005). In mice, *MECP2* overexpression causes hypoactivity and seizures (Collins et al. 2004; Bodda et al. 2013). The amount of *MECP2* required to cause toxicity associated with overexpression has been debated, with some studies finding that 1.6 × normal levels of *MECP2* cause behavioral impairments in mice (Jugloff, 2008) and others finding that 2.4 × normal *MECP2* can be tolerated (Koerner et al. 2018). Despite this discrepancy, small deviations in *MECP2* levels in humans have been linked to milder neurological and psychiatric conditions including autism, intellectual disability, and lupus erythematosus. Thus, caution must be taken to provide enough *MECP2* per cell to impart a therapeutic benefit while reducing the risk of *MECP2* overexpression.

### Read-through compounds may facilitate the recovery of *MECP2* product

One strategy involves directly targeting the *MECP2* mutation with small molecules known as translational read-through-inducing drugs (TRIDs). This is an attractive strategy for 35–40% of Rett syndrome patients who have nonsense mutations in *MECP2* that result in premature stop codons, which inhibit normal full-length protein expression (Neul et al. 2008). TRIDs permit read-through of premature stop codons by binding to ribosomes and impairing codon/anticodon recognition, allowing for the insertion of another amino acid in place of the stop codon (Nagel-Wolfrum et al. 2016). Notably, this causes a missense mutation rather than a nonsense mutation, which may impart less of a functional consequence on the protein product. Importantly, this strategy would increase *MECP2* in cells with the nonsense mutation, while avoiding increasing dosage in cells with normal *MECP2*. However, because TRIDs would create a mutated *MECP2* rather than a fully functional protein, this treatment strategy may only modestly improve symptoms in patients. An additional concern is nonsense mediated decay (NMD), a cellular process that facilitates the degradation of mRNA transcripts with premature stop codons because their translation could lead to mutated proteins with deleterious gain-of-function or dominant-negative effects (Conti and Izaurralde 2005). In mammalian cells, when a premature stop codon is located greater than 50–55 nucleotides away from an exon–exon junction, NMD is activated (Nagy and Maquat 1998; Kuzmiak and Maquat 2006). NMD naturally reduces the number of transcripts available for TRIDs (Linde et al. 2007). Therefore, TRIDs may not be a suitable treatment option for RTT patients with certain nonsense mutations which are prone to NMD as the best responders will have high levels of target transcript. However, the efficacy of NMD varies naturally between individuals (Nguyen et al. 2014) and patient transcript levels could be assayed to determine if they are candidates for TRID therapy.

One class of TRIDs is aminoglycoside antibiotics, of which gentamicin is the most commonly studied. Gentamicin has been shown to suppress nonsense mutations and restore function protein in mouse models of Duchenne muscular dystrophy (DMD) (Barton-Davis et al. 1999), cystic fibrosis (CF) (Du et al. 2008), retinal degeneration (Guerin et al. 2008), and several other diseases. Importantly, clinical trials in DMD and CF patients have demonstrated gentamicin’s ability to restore protein products in a subset of patients, giving hope for the effectiveness of TRIDs in other diseases (Nagel-Wolfrum et al. 2016).

Four of the most common RTT-causing mutations (R168X, R255X, R270X, R294X) result in premature stop mutations. In vitro studies have shown that read-through efficiency of gentamicin depends heavily on the context of the stop mutation, where gentamicin restored 22% of function *MECP2* in cells with R294X mutations and only 10% in cells with R168X (Brendel et al. 2011), possibly due to variable NMD. Increasing the concentration of gentamicin increased read-through efficiency and amounts of *MECP2* protein. However, these high levels of aminoglycosides could cause toxic side effects if administered over a long period of time (Popescu et al. 2010). Novel aminoglycosides and different TRIDs may impart higher read-through activity and lower toxicity (Brendel et al. 2011; Pitcher et al. 2015), though no in vivo tests with these novel compounds have performed in *Mecp2*-mutant mice.

One new promising TRID is Ataluren, an orally bioavailable oxadiazole with no antibacterial activity and minimal off-target effects (Keeling et al. 2014). Ataluren restored 20% of normal functional protein in mouse models of DMD and CF, leading to clinical trials for both diseases. However, in both cases, Ataluren did not produce enough protein to see a significant benefit in patients (Kerem et al. 2014; McDonald et al. 2017). This may be a worthwhile avenue to pursue for RTT since even small increases in *MECP2* are associated with improved symptoms. Nonetheless, efforts are underway to improve the efficacy of TRIDs for suppression therapy, making them more likely to assist RTT patients.

### Reactivating the silent X chromosome could restore endogenous *MECP2* to cells

Through XCI, mammalian cells silence one X chromosome in each cell, allowing for dosage compensation of X-linked genes (Bhatnagar et al. 2014). XCI is initiated by the non-coding RNA *Xist*, which coats the inactive X chromosome (Xi) from which it is produced. On the active X (Xa), *Tsix* RNA blocks *Xist* upregulation. As *MECP2* is expressed on the X chromosome, one copy is silenced by XCI. Therefore, in cells of RTT patients where the mutated *MECP2* is on the Xa, a normal copy of *MECP2* lies dormant on the Xi.

Interestingly, the silent state of the Xi is temporarily reversed during development and stem cell remodeling. In the case of X-linked diseases, identification of key molecular players involved in reactivation of the Xi could be repurposed to facilitate the re-expression of its genes. Using this strategy to target *MECP2* could provide a viable treatment option for RTT patients (Vacca et al. 2016). This is an attractive strategy for RTT because patient cells could use their own regulatory functions to control *MECP2* expression. However, sex chromosome dosage is an important developmental process and reactivating the second X chromosome may lead to a pathological level of expression for other X chromosome loci. Ideally, these strategies will aim to target the *MECP2* gene or its local vicinity alone. Additionally, the Xi can be difficult to reactivate due to multiple mechanisms of epigenetic silencing and attempting to disengage these processes could disrupt epigenetic patterns throughout the genome which could cause adverse long-term effects. Finally, because RTT patients are heterozygous mosaics for *MECP2* mutation, approximately half of their cells already express a normal copy of *MECP2*. Thus, Xi reactivation will lead to 2x the normal amount of *MECP2* in these cells. In patients carrying mutations that only reduce efficiency of *MECP2* rather than destroy its function, Xi reactivation will lead to anywhere from 1 to 2x the normal amount of *MECP2* in some cells. This could lead to symptoms associated with overexpression of *MECP2*. Therefore, Xi reactivation will need to be adapted to target only *MECP2* or its local vicinity, and may only be a practical treatment option for patients who have loss-of-function mutations in *MECP2*.

One group reactivated the Xi using an antisense oligonucleotide to 5-aza-2′-deoxycytidine (Aza), a DNA methylation inhibitor, in a mouse with an *Xist* deletion on one chromosome and *Mecp2*-GFP on the other. This ensured that *Mecp2-*GFP was silenced in almost all cells. Treatment resulted in a great increase in GFP expression, indicating that *Mecp2* could be targeted by Xi reactivation tools (Carrette et al. 2017). Recently, a heterozygous female mouse was engineered with a mutation of *Tsix* on one chromosome and a null mutation of *Mecp2* on the other, directing the preferential expression of the chromosome with the mutated *Mecp2* (Carrette et al. 2018). This strategy achieved a *Mecp2*-null female mouse with all cells expressing the same Xa. Additionally, this group found that while *Mecp2*-null females had a shortened lifespan, a 5% increase in *MECP2* levels increased the lifespan by 50%, and a 10–20% increase in *MECP2* restored a normal lifespan (Carrette et al. 2018). Thus, small increases in *MECP2* can have great positive impacts on life expectancy. However, as a global inhibitor of methylation, Aza is toxic when administered over a long time course. Ongoing screens aim to identify new molecules to induce Xi reactivation that can be further tested and optimized for treatment (Bhatnagar et al. 2014; Minkovsky et al. 2015; Sripathy et al. 2017). Importantly, the unique mice designed in these studies will provide an excellent avenue to test candidate Xi-reactivating drugs specifically for the treatment of RTT.

### Gene therapy

A final potential avenue of treatment for RTT involves introducing a normal copy of *MECP2* into cells by gene therapy. Gene therapy is a promising approach for the treatment of many disorders and has been successful in reversing symptoms in mouse models of CF, hemophilia, Hunter syndrome, diabetes, obesity, ALS, and more. Recently, the first targeted gene therapy treatment was approved in North America to treat patients with Lever’s congenital amaurosis, a rare inherited eye disease (Kumaran et al. 2018).

To treat RTT, gene therapy approaches must utilize an appropriate vector able to cross the BBB and transduce many cells in the CNS, and able to maintain stable long-term expression of the exogenously derived *MECP2*. Additionally, strategies must be developed to avoid transgene repression and avoid overexpression of *MECP2*. Like Xi reactivation, gene therapy targets all cells regardless of *MECP2* mutation status. Thus, cells expressing normal *MECP2* will have 2x the normal level of the protein leading to potential toxic effects of *MECP2* overexpression. An additional concern of gene therapy is that high viral titres are often needed to infect a large proportion of cells, but cells that inadvertently receive more than one viral particle, and hence more than one copy of *MECP2*, would be overburdened. Strategies to circumvent these issues could supply a factor such as an siRNA to suppress endogenous *MECP2* so that only the transgenic *MECP2* is expressed (Gadalla et al. 2011). However, this would require the transgenic *MECP2* to possess a seamless promoter to completely mimic endogenous gene expression. MicroRNAS (miRNAs) have been used to control exogenous transgene expression by mediating the degradation of transgene mRNAs in a tissue-specific manner and may be beneficial in RTT gene therapy approaches to limit off-target toxicity (Geisler and Fechner 2016).

Recombinant adeno-associated virus (AAV) vectors have been used in preclinical gene therapy studies for their ability to cross the BBB, infect neurons, and mediate stable long-term expression of the transgene without inflammation or toxicity (Gonçalves 2005; Foust et al. 2009). AAV has a 4.7-kb ssDNA genome from which 4.4 kb of the viral DNA can be removed and replaced with a human transgene. Self-complementary (sc) AAV vectors have a 10–100-fold higher transduction efficiency, but their drawback is that their packaging capacity is cut in half to only 2.2 kb, making it difficult to package large genes (McCarty et al. 2001). When AAV9-*MECP2* under the promoter of chicken β-actin was injected into the brains of neonatal *Mecp2-*null mice, transduction efficiency varied between brain regions from 7 to 42% of cells infected, with the highest infection rate in the hypothalamus and lowest in the striatum (Gadalla et al. 2013). However, this low efficiency of infection was sufficient to increase lifespan of male mice to 16.6 weeks and improve motor impairments, but it did not have any effect on respiratory symptoms. In contrast, scAAV9-*MECP2* under a truncated *Mecp2* promoter injected into mice systemically had a very low transduction efficiency in the brain of 2–4%. Despite this, mice survived to 15 weeks, indicating that low levels of *MECP2* re-expression in the brain and/or re-expression in non-CNS tissues could modestly improve lifespan. Notably, AAV9 preferentially targeted the liver and spleen, with some cells in these tissues receiving ten copies of the vector and causing liver damage. Therefore, future studies must increase the specificity of AAV9 therapy before moving to the clinic.

A subsequent study delivered scAAV9-*MECP2* under a truncated *Mecp2* promoter systemically and found a range of transduction efficiencies in the brain from 10% in the cerebellum to 25% in the cortex and brainstem (Garg et al. 2013). This produced some behavioral improvements but did not rescue breathing symptoms. A second-generation AAV9 vector with a modified 3′UTR and a panel of miRNA binding sites was developed with the goal of biasing transgene expression away from the liver (Sinnett et al. 2017). This vector, injected in the cisterna magna, was better tolerated and improved the lifespan of *Mecp2* mutant mice but could not improve behavioral traits without being used at very high doses. However, direct cerebroventricular injection of this vector into neonatal *Mecp2-*null mice resulted in a high brain transduction efficiency, increased survival, and the amelioration of RTT-like phenotypes, highlighting the importance of endogenous regulatory elements in the gene expression cassette (Gadalla et al. 2017).

Finally, a recent study designed a minimal-*MECP2* protein lacking all amino acids except those encoding two functional domains: the methyl-binding domain, and the NCOR-interaction domain (Tillotson et al. 2017). When neonatal mice were injected intracranially with a scAAV9 vector encoding this minimal protein, they showed improved phenotypes and survival in the absence of toxic effects, indicating that the primary role of *MECP2* is to physically connect the NCOR-containing complex to DNA. This study provides a new avenue to pursue in gene therapy studies as a functional minimal *MECP2* protein creates room for additional regulatory sequences to be packaged into the limited capacity of scAAV9 vectors. Importantly, this would allow for more precise temporal control of *Mecp2* expression. Future studies should aim to introduce additional regulatory elements into the gene therapy vector while also controlling for timing of treatment to best represent therapeutic utility in human patients.

An additional obstacle of gene therapy treatment for any disorder involves scaling the dosage for humans. Due to their small size, mice cannot reliably inform effective dosages for clinical applications. Therefore, gene therapy treatment is being tested in other animal models including canines and non-human primates. The brains of non-human primates are similar to humans with regard to neural circuitry, physiology, and behavioral characteristics, making them an ideal model to test gene therapy for neurological diseases (Gopinath et al. 2015). However, studies in larger models are expensive and require longer experimental periods. Importantly, primate models lacking *MECP2* have been generated which can be utilized to accelerate gene therapy treatment for RTT (Liu et al. 2014; Chen et al. 2017). Recently, systemic administration of high-dose AAV9 was found to cause severe liver and neuronal toxicity in three non-human primates indicating that efforts in dosage optimization will be imperative (Hinderer et al. 2018).

## The need for precision medicine for a monogenic disease

Rare diseases have limited statistical power in clinical trials making animal models our greatest opportunity at developing, adapting, and enhancing therapeutics to be clinical trial-ready. Importantly, treatments that are rigorously tested and streamlined for efficiency before advancing to human trials achieve higher levels of success than those for which multiple preclinical trials are not conducted (Morgan et al. 2018). To do this, studies should address age- and method-dependent effects of treatments. For example, in the case of gene therapy, treatment is often more effective in neonatal mice compared to adult mice, or through direct brain injection compared to systemic administration. It is likely that most treatment options will be more effective if delivered to patients early, but as this is not always feasible, preliminary studies should aim to recapitulate treatment methods available in human patients. This would involve testing treatments in *Mecp2* mutant mice after symptom onset. Additionally, identifying and publishing negative effects of potential therapeutics is beneficial as they inform about off-target effects and toxicity. This ultimately provides better criteria to assess the safety of treatments that will lead to the development of more effective therapeutic strategies.

While many potential treatments for RTT have been identified in model organisms, many have not shown extreme efficacy in human trials. Clinical trial design for rare diseases is especially important due to the sparsity of patients. In diseases like RTT where there is substantial variability in clinical presentation, patient stratification should be considered to reduce the variability in the sample and maximize the potential to detect efficacy (Modi and Sahin 2018). Natural history studies assessing genotype–phenotype correlations, symptom progression, and biomarkers of disease may provide tools to identify patients likely to respond to new treatments. For example, there may be critical windows in which specific interventions will have a maximal effect. Additionally, clinical trials should be designed with enough statistical power for the endpoints being tested, with consideration given to placebo effects.

**Fig. 4:**
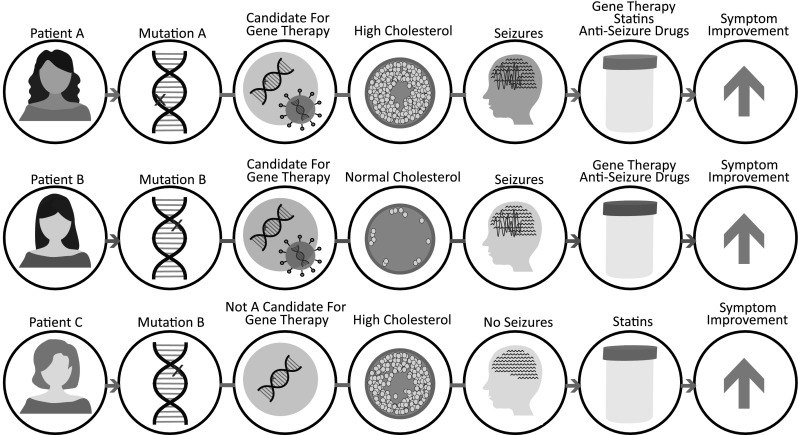
Precision medicine for RTT. Different RTT patients will likely benefit from different combinations of treatment. When treating patients for RTT, their mutation status and XCI skewing should be taken into consideration. It is likely some patients will not be ideal candidates for gene therapy. Biomarkers, such as serum cholesterol, can be used to determine which patients will benefit from pharmacological intervention, such as statins. Management of other symptoms (seizures, scoliosis, lung infection, etc.) should also be considered. Together, RTT patients should receive individualized treatment to maximize their symptom improvement

Given the drastic variation in phenotype presentation, it is likely that every RTT patient is unique and will not respond to the same treatment. In this respect, precision medicine for RTT is warranted (Fig. 4). Precision medicine approaches are taken because patients carrying different mutations do not always respond to treatments in the same way. Ideally, RTT patient age, their specific mutation and individual level of XCI skewing should be taken into consideration when developing a personalized treatment regimen. It is likely that some patients will make excellent candidates for gene therapy, mutation read-through, or reactivation of the silent X chromosome, while others may not benefit greatly from any of these treatments.

As CNS-targeted gene therapy becomes a more realistic therapeutic approach, peripheral deficiency of *MECP2* also needs to be considered. Loss of *MECP2* in non-CNS tissues is associated with cardiac defects (Hara et al. 2015), decreased bone density (Haas et al. 1997), urological dysfunction (Ward et al. 2016), elevated lipid metabolism (Justice et al. 2013; Segatto et al. 2014), and increased oxidative stress (Leoncini et al. 2011). It is likely that when *MECP2* is genetically treated exclusively in the brain, these peripheral symptoms will persist. For this reason, biomarkers of non-CNS perturbations in RTT are needed to ensure adequate treatment. For example, high serum cholesterol or increased oxidative stress can be used as an indicator for statin or triheptanoin treatment, respectively. As more perturbed pathways downstream of *Mecp2* mutation are discovered, it is likely that the list of biomarkers for pharmacological intervention will increase. Importantly, patients should continue to be assessed for seizures, scoliosis, lung infection, and other features of RTT that present differently in each patient. Ultimately, because small increases in *MECP2* can lead to drastic improvements (Carrette et al. 2018), it is probable that a combination of gene therapy paired with personalized pharmacological treatments will provide the best efficacy to treat most RTT patients. Treating each patient individually is expensive which can reduce profit for drug companies. However, the NIH instituted an orphan drug program to provide incentives for pharma companies to develop treatments for rare diseases such as RTT (Engstrom 1985).

Although it is a rare disorder, RTT has served as a paradigm for the understanding and developing treatments for rare diseases because of the early development of mouse models, intensive mechanistic studies, a NIH-funded natural history study (NCT02738281), and funding by focused patient groups (International Rett Syndrome Foundation and Rett Syndrome Research Trust). Because it is a complex disorder involving many organ systems, it is likely that recovery of RTT patients will involve a combination of treatments. Model organisms will continue to serve as an indispensable tool in accelerating drug discovery and adapting therapeutics, leading to better treatment strategies for many rare disorders.
